# miR-202 functions as a tumor suppressor in non-small cell lung cancer by targeting STAT3

**DOI:** 10.3892/mmr.2022.12811

**Published:** 2022-08-02

**Authors:** Zhonghai Zhao, Bin Lv, Li Zhang, Nana Zhao, Yan Lv

Mol Med Rep 16: 2281–2289, 2017; DOI: 10.3892/mmr.2017.6841

Following the publication of this paper, it was drawn to the Editors’ attention by a concerned reader that certain of the cell migration assay data shown in Fig. 2C were strikingly similar to data that had appeared in different form in another article by different authors. Owing to the fact that the contentious data in the above article had already been published elsewhere prior to its submission to *Molecular Medicine Reports*, the Editor has decided that this paper should be retracted from the Journal. The authors were asked for an explanation to account for these concerns, but the Editorial Office did not receive a reply. The Editor apologizes to the readership for any inconvenience caused.

